# Unraveling the *Catha edulis* Extract Effects on the Cellular and Molecular Signaling in SKOV3 Cells

**DOI:** 10.3389/fphar.2021.666885

**Published:** 2021-05-10

**Authors:** Alaa Sayed Abou-Elhamd, Gauthaman Kalamegam, Farid Ahmed, Mourad Assidi, Abdulmajeed Fahad Alrefaei, Peter Natesan Pushparaj, Muhammad Abu-Elmagd

**Affiliations:** ^1^ Department of Anatomy and Histology, Faculty of Veterinary Medicine, Assiut University, Assiut, Egypt; ^2^ Department of Respiratory Therapy, College of Applied Medical Sciences, Jazan University, Jazan, Saudi Arabia; ^3^ Department of Medical Laboratory Technology, Faculty of Applied Medical Sciences, King Abdulaziz University, Jeddah, Saudi Arabia; ^4^ Center of Excellence in Genomic Medicine Research, King Abdulaziz University, Jeddah, Saudi Arabia; ^5^ Department of Biology, Jamoum University College, Umm Al-Qura University, Mecca, Saudi Arabia

**Keywords:** molecular signaling, gene expression, FGF, microRNAs, khat, SKOV3

## Abstract

Khat (*Catha edulis* (Vahl) Endl.) is an evergreen flowering shrub used as a stimulant in many regions worldwide including East Africa, the Arabian Peninsula, Europe, and the United States. Chewing leaves of khat induces excitement and euphoria, which are primarily attributed to two major constituents, cathinone and cathine. Khat also contains other important constituents such as cathedulins. A considerable number of studies reported side effects induced by the khat extracts to both embryos and adults. These include teratogenicity and developmental retardation, oral cancer and ulcers, high blood pressure, and myocardial infarction. So far, little attention has been paid to the effects of khat extracts on the molecular signaling interactions. We aimed in this study to investigate this through evaluating the effects of khat extracts on SKOV3, a human ovarian adenocarcinoma cell line. We show, by *in vitro* assays, that khat induces several cellular defects including reduced cell size, cell membrane damage, and apoptosis. At high khat extract concentrations, the cell metabolic activity, cell cycle, and cellular proliferation were affected. RT-qPCR analysis showed an increase in the gene expression of the apoptotic marker BAX, the tumor suppressor p53, and the inflammatory cytokine IL-6. Protein expression analysis by immunostaining showed downregulation of β-catenin, E-cadherin, and Ki-67 and upregulation of FZD8 and SPRY2, suggesting that Wnt and FGF signaling were implicated. SwissTargetPrediction *in silico* analysis showed that khat constituents cathine, cathinone, catheduline K2, and catheduline E5 bind to family A G-protein-coupled receptor, cause many neurological diseases and disorders such as Alzheimer's, schizophrenia, depression, and anxiety, and induce many ovarian cancer-related diseases. The analysis also showed that important signaling pathways such as CREB, Wnt, FGF, IL-6, and ERK/MAPK, and that of the endometrial cancer, and cell cycle were implicated. Upstream regulators of cathine and cathinone were found to potentially target several molecules including interleukin-8, MMP2, PLAU, and micro-RNAs. In conclusion, khat induces significant cellular and molecular changes that could potentially cause a wide range of serious diseases and syndromes. Such an impact could have a heavy burden on the health care system in the countries where khat is consumed.

## Introduction

Khat (*Catha edulis* (Vahl) Endl.) is an evergreen shrub grown in the Horn of Africa and the Arab Peninsula (Al-Qirim et al., 2002; Wabe, 2011). Chewing khat leaves is a common practice among people in these countries due to its stimulant and sympathomimetic effects (Al-Qirim et al., 2002; Dimba et al., 2004; Al-Zubairi et al., 2008). At least 200 constituents in the khat leaves have been recently identified (Kiros, 2020). Khat leaves contain a considerable number of alkaloids, among which major active constituents are cathinone (S-(-)-2-amino-1-phenyl-1-propanone), cathine (1S, 2S-norpseudoephedrine), 62 highly complex cathedulins (polyhydroxylated sesquiterpenes), flavonoids, glycosides, ascorbic acid, tannins, sterols, triterpenes, and smaller amounts of 1*R*, 2*S*-norephedrine (Halbach, 1972; Kalix and Braenden, 1985; Kite et al., 2003; Wabe, 2011; Alsanosy et al., 2020; Kiros, 2020). Young and mature khat leaves constituent analysis has been recently well-documented. This hierarchical cluster analysis showed that cathine and cathinone were the major components and associated with significant cytotoxicity (Alsanosy et al., 2020). The use of khat for medicinal treatment is not yet fully understood, but it has been used to treat serious diseases such as gonorrhea, asthma, chest complications, depression, gastric ulcers, obesity, and tiredness (reviewed by Kiros, 2020).

Problems associated with repeated khat consumption such as psychiatric morbidity (Al-Habori et al., 2002; Pennings et al., 2008), myocardial infarction (Al-Motarreb et al., 2002a), muscle toxicity, and rhabdomyolysis (Mohan et al., 2019), as well as hypertension and tachycardia (Kuczkowski, 2005) have been evident. Khat also causes adverse effects on the gastrointestinal tract such as esophagitis and gastritis (Gunaid et al., 1995). Khat intake has been shown to have a direct relationship with oral cancers with signs of cytotoxic effects at the site of mastication (Soufi et al., 1991; Ali et al., 2004). Interestingly, the evidence for the direct link between khat consumption and oral cancer induction has recently shown a great deal of controversies (Al-Maweri et al., 2020; Chong et al., 2020). Some khat extracts cytotoxic effects were reported in ovarian cancer (Elhag et al., 1999). The toxicity symptoms caused by heavy khat consumption were attributed to the harmful effects of its polyphenolic contents (Abdelwahab et al., 2018).

In rats, khat extracts induced apoptosis, degeneration of hepatocytes (Al-Habori et al., 2002; Al-Mamary et al., 2002; Ageely et al., 2014), embryotoxicity, and severe teratogenicity (Islam et al., 1994; Abou-Elhamd et al., 2018). In male rats, it impaired the process of spermiogenesis (Abou-Elhamd et al., 2020), while in females, it increased the oxidative stress markers (Arafa et al., 2019).

At the cellular level, khat causes genetic damage in human T-lymphoblastoid cell lines (Barkwan et al., 2004) and rapid cell death in HL-60, Jurkat, and NB4 leukemia cell lines (Dimba et al., 2003).

Literature evidence from both *in vitro* and *in vivo* studies indicated that khat extracts have significant cytotoxic and apoptotic effects against some human cancer cell lines (Dimba et al., 2003; Bredholt et al., 2009; Alsanosy et al., 2020). Ovarian cancer ranks fifth most common among cancers in women and is associated with high morbidity and mortality. SKOV3 is an epithelial-like human ovarian adenocarcinoma cell line derived from the ascites of an ovarian serous cystadenocarcinoma. To understand whether khat extract has any inhibitory or cytotoxic effects on ovarian cancer cells, we, in the present study, evaluated the effects of khat extracts on SKOV3 using both *in vitro* and *in silico* platforms. We showed significant changes in the gene and protein expression of several known molecular markers. We also identified many signaling pathways implicated by the khat extracts such as FGF signaling, Wnt signaling, and micro-RNAs.

## Materials and Methods

### Ethical Approval

The ethical approval for the use of SKOV3 was obtained from the Bioethics Committee of the King Abdulaziz University *vide* approval number (33-15/KAU). Khat extracts were processed and obtained from the Biology Department, Jazan University, and its use was approved by the Research Ethics Committee of the Medical Research Center, Jazan University (approval number REC/ MRC/ JU, 30/01/2017).

### Khat Extraction

Two bundles weighing 393 g of fresh khat (genus: *Catha edulis* (Vahl) Endl., family: Celastraceae, order: Celastrales, class: Magnoliopsida) was provided by the Substances Abuse and Research Center, Jazan University, Jazan, Saudi Arabia, on the 12th of January 2019. Khat extraction was carried out as soon as the khat bundles were received to avoid the loss of the active ingredients. The average length of the selected khat green leaves for the extract preparation was 4–6 cm. Khat leaves (126 g) were washed in distilled water, chopped on a metal plate, and crushed by a blender. Methanolic khat extraction was carried out as previously described (Abou-Elhamd et al., 2020). In brief, crushed leaves were immersed in 100 ml methanol (Sigma-Aldrich, Taufkirchen, Germany) and kept on a rotary shaker for 2 h. The mixture was filtrated through an 11 mm filter paper (Grade 1, Whatman, Kent, United Kingdom). The filtrate was kept overnight on a stirrer at 45°C to allow the methanol to evaporate (Kimani et al., 2008). Dried khat methanolic crude extract weighed 65.5 g from which we obtained approximately 34 g of usable khat extracts. Cathine and cathinone concentrations in the khat extracts were measured in the Poison Control Medicinal Chemistry Legitimacy Jazan Center, Jazan, Ministry of Health, Saudi Arabia, and were 305 μg/ml and 114 μg/ml, respectively. Khat extracts were stored at −20°C until used as required. Khat extracts were solubilized before being used in the experiments by a tissue culture grade phosphate-buffered saline without calcium and magnesium (PBS^−^) and sonicated for 3 min (50 Hz, 37°C).

### Culture of the Human Ovarian Cancer Cell Line (SKOV3)

SKOV3 was purchased from the European Collection of Authenticated Cell Cultures (ECACC, Wiltshire, England). The SKOV3 cells were cultured in the basal Rosewell Park Memorial Institute (RPMI) 1640 medium supplemented with 10% fetal bovine serum (FBS), 2 mM GlutaMAX, and 1% antibiotics (penicillin-streptomycin). The frozen vials containing SKOV3 cells were rapidly thawed in 37°C water bath using the standard thawing procedure. The cells were cultured in a humidified 5% CO_2_ incubator at 37°C.

### Cell Morphology

SKOV3 cells were seeded at a density of 2 × 10^4^ cells/well in a 24-well plate and allowed to attach overnight. SKOV3 cells serving as negative controls were treated with PBS^−^ with volumes equal to the added khat extracts. The cells were then treated with various concentrations of the khat extracts (10 μg/ml, 30 μg/ml, 100 μg/ml, 300 μg/ml, 1 mg/ml, 3 mg/ml, and 10 mg/ml) for 24–72 h and cultured in a 5% CO_2_ incubator at 37°C. Cell morphology was investigated every 24 h by phase-contrast microscopy (Nikon Instruments, Tokyo, Japan).

### Cell Metabolic Activity (MTT) Assay

SKOV3 cells were plated and cultured as stated above, and MTT assay was carried out at 24, 48, and 72 h following treatment with khat extracts at different concentrations (10 μg/ml, 30 μg/ml, 100 μg/ml, 300 μg/ml, 1 mg/ml, 3 mg/ml, and 10 mg/ml). The cell metabolic activity and hence their proliferation/inhibition was determined using MTT assay according to the manufacturer’s instructions (Sigma-Aldrich, Germany). The spun media was removed, and 200 ml of fresh culture medium containing 20 µl MTT reagent (3-(4, 5-dimethyl thiazolyl-2)-2, 5-diphenyltetrazolium bromide; Sigma, MO, United States) was added to each well. The cells were incubated under standard culture conditions for 4 h. The medium was removed, and the insoluble formazan crystals were solubilized using DMSO (200 ml/well). The absorbance was obtained at 570 nm with a background reference of 630 nm, using a SpectraMax i3 Multimode Reader (Molecular Devices, Sunnyvale, CA, United States).

### Cell Cycle Assay

SKOV3 cells were seeded at a density of 1 × 10^5^ cells/T-25cm^2^ tissue culture flask and treated with khat extracts at the following concentrations: 10 μg/ml, 30 μg/ml, and 300 μg/ml for 48 h. The control and treated cells were fixed in 70% ice-cold ethanol by dropwise addition, to avoid clumping of cells, and left overnight at 4°C. The fixed cells were then washed with PBS^−^ and stained with propidium iodide (PI, 50 μg/ml) in PBS^−^ containing 50 μg/ml RNase-A and 0.1% Triton X-100. The cells were analyzed using a FACS III Aria flow cytometer (BD Biosciences, CA, United States), and the results were computed with FACSDiva™ software (BD Biosciences, CA, United States).

### Apoptosis (Annexin V-PI) Assay

SKOV3 cells were plated and treated with khat extracts as in the above experiment. Cells were treated with khat extracts at the following concentrations: 10 μg/ml, 30 μg/ml, and 300 μg/ml for 48 h. Both the control and treated cells were then trypsinized, centrifuged (1000 rpm × 5 min), and pelleted. The cell pellet was washed once in cold PBS^−^ and twice in 1X binding buffer with centrifugation (1000 rpm × 5 min) in between washing steps. The final cell pellet was resuspended in freshly prepared PI (Sigma, St Louis, MO, United States) and Annexin V-APC (BD Biosciences, CA, United States) solution and incubated for 15 min in the dark at room temperature. The stained samples were then analyzed for the various stages of the apoptotic cells using a FACS III Aria flow cytometer (BD Biosciences, CA, United States), and the results were computed with FACSDiva™ software (BD Biosciences, CA, United States).

### Gene Expression Analysis (Quantitative Real-Time PCR)

SKOV3 cells were treated with khat extracts as mentioned above and were analyzed for the apoptosis, cell cycle, and inflammation-related gene expression using quantitative real-time PCR (RT-qPCR). The total RNA was extracted using a Pure Link RNA Mini Kit (Ambion, Thermo Fisher Scientific, United Kingdom). Quantity and quality were analyzed using Nanodrop™ (Nanodrop Technologies, Wilmington, DW, United States). First-strand cDNA synthesis was done using random hexamers (High-Capacity cDNA Reverse Transcription Kit, Applied Biosystems) with the inclusion of on-column deoxyribonuclease (DNase-I) treatment. Gene expression of BAX (apoptosis regulator, also known as BCL-2-like protein 4), p53 (tumor suppressor), and IL-6 (interleukin, acts as a proinflammatory cytokine and an anti-inflammatory myokine) were analyzed. The primer sequences used are provided in Table 1 (Abu-Elmagd et al., 2017). Gene expression analysis was performed using a StepOnePlus^TM^ real-time PCR System (Applied Biosystems, United States) with SYBR Green Master Mix. Relative quantitation was done using the comparative 2^−ΔΔCt^ method.

**TABLE 1:** Genes and primers’ sequence details.

Gene	Primer sequence
BAX	F: 5′-TGG​AGC​TGC​AGA​GGA​TGA​TTG-3’
	R: 5′-GCT​GCC​ACT​CGG​AAA​AAG​AC-3′
IL6	F: 5′-CCA​CTC​ACC​TCT​TCA​GAA-3′
	R: 5′-GCG​CAA​AAT​GAG​ATG​AGT-3′
p53	F: 5′-GCG​CAC​AGA​GGA​AGA​GAA​TC-3’
	R: 5′-CTC​TCG​GAA​CAT​CTC​GAA​GC-3′
GAPDH	F: 5′-ACC​ACA​GTC​CAT​GCC​ATC​AC-3′
	R: 5′-TCC​ACC​ACC​CTG​TTG​CTG​TA-3′

### Immunohistochemistry

SKOV3 control cells and 700 μg/ml of khat extract-treated cells on coverslips were fixed in 4% formaldehyde/PBS for 10 min at room temperature and then rinsed twice in ice-cold PBS/Tween-20 (PBST). Cells were then treated with 2% H_2_O_2_ for 10 min to block the endogenous peroxidase expression, washed with PBST, and then permeabilized with Triton X-100 for 10 min. Cells were washed with PBST and blocked with 10% goat serum, after which each coverslip was treated at 4°C with an anti-human primary antibody of β-catenin (Leica Biosystems, IL, United States) (mouse monoclonal, 6003258, 1:100 dil.), E-cadherin (Dako, CA, United States) (mouse monoclonal, M7240, 1:100 dil.), Frizzled-8 (FZD8, Rabbit, ab155093, 1:100 dil.), Sprouty-2 (SPRY2, mouse monoclonal, SC-100862, 1:250 dil.), and Ki-67 (mouse monoclonal, M-7240). After overnight incubation in the primary antibody, cells were washed with PBST and blocked with 10% goat serum. The secondary antibody was applied, and the color was detected according to Dako REAL detection system manufacturer’s instructions (Dako, CA, United States, cat. no. K5001). Cells were then treated with biotinylated secondary antibody for 20 min, washed with PBST, and then treated with streptavidin peroxidase for 20 min. The color was developed using Dako DAB color substrate and counter-stained with hematoxylin. The slides were dehydrated with ascending grades of ethanol and mounted with xylene-based mounting media. Images were captured using an Olympus BX53 microscope (Tokyo, Japan).

### *In Silico* Analysis

#### SwissTargetPrediction

The machine-readable formats of the cathine (C_9_H_13_NO), cathinone (C_9_H_11_NO), catheduline K2 (C_40_H_51_NO_19_), and catheduline E5 (C_59_H_64_N_2_O_23_) structures were obtained, based on both canonical and from the PubChem Database (Kim, 2016; Kim et al., 2016). In the present study, the putative molecular targets of the cathine, cathinone, and both cathedulins were obtained using SwissTargetPrediction (http://www.swisstargetprediction.ch/) (Gfeller et al., 2014) (Supplementary Figure S1). Canonical and isomeric SMILES of cathine, cathinone, catheduline K2, and catheduline E5 were used as input sequences in the SwissTargetPrediction webserver to virtually screen the molecular targets (Daina and Zoete, 2019). The SwissTargetPrediction virtual screening tool uses the "similarity principle" to predict the most probable targets of bioactive molecules such as cathine, cathinone, catheduline K2, and catheduline E5 (Gfeller et al., 2013; Gfeller et al., 2014). In this virtual reverse screening tool, the putative binding predictions are accomplished from 376,342 experimentally active analogous compounds in 2D and 3D that strongly interact with 3,068 well-recognized protein targets (Huang et al., 2018; Daina and Zoete, 2019)**.** In the latest version of the SwissTargetPrediction, the dataset is based on ChEMBL23, and putative protein targets are ranked based on a score that merges both 2D and 3D similarity values of an active molecule to the query molecules such as cathine, cathinone, catheduline K2, and catheduline E5 (Daina et al., 2019). Importantly, the ranking of the targets rather than the absolute values of scores or probabilities is the most meaningful parameter. A maximum of 100 probable protein targets was obtained as an output from the SwissTargetPrediction tool (Gfeller et al., 2014; Daina et al., 2019).

#### WebGestalt Analysis of Cathine and Cathinone Targets

WebGestalt (wGSEA) is an open-source platform (http://webgestalt.org/) that facilitates a more flexible, comprehensive, and interactive functional enrichment analysis of differentially expressed proteins (DEPs) or differentially expressed genes (DEGs) (Liao et al., 2019). The newest version of the wGSEA identifies 155,175 functional groups, 342 gene identifiers, and 12 major organisms with an additional option for including user-defined functional databases (Liao et al., 2019; Bahlas et al., 2020). The DEPs or DEGs derived from medium- to large-scale omics experiments can be classified based on biological, molecular, and cellular functions using the wGSEA web tool. To functionally classify the cathine- and cathinone-induced putative protein targets, the Over Representation Analysis (ORA) module of the wGSEA was chosen (Supplementary Figure S1 **)**, the preferred organism was *Homo sapiens*, and gene ontology (biological, cellular, and molecular functions) and disease databases such as OMIM, GLAD4U, and DisGeNET were selected for further downstream analyses (Liao et al., 2019; Bahlas et al., 2020). The default parameters for the enrichment analysis such as the minimum number of IDs (5), the maximum number of IDs (2000), the Benjamini Hochberg (BH) method for computing the False Discovery (FDR) Rate (*p* < 0.05), and the significance level (Top 10) were applied for each wGSEA analysis (Bahlas et al., 2020).

#### Open Targets Platforms

The Open Targets Platform (https://www.targetvalidation.org/) was utilized to uncover the cathine and cathinone molecular targets associated with cell proliferation disorders, ovarian diseases, and psychiatric disorders (Koscielny et al., 2017; Carvalho-Silva et al., 2018; Ochoa et al., 2021). The evidence from various omics studies, text mining of scientific publications, *in vivo* models, and disease relevant drugs were utilized in the Open Targets Platform to score and rank target-disease associations and assist target prioritization (Khaladkar et al., 2017; Carvalho-Silva et al., 2018; Ochoa et al., 2021). Here, the query lists, along with the putative molecular targets of cathine and cathinone, were used to decipher the cell proliferation, ovarian, psychiatric, and nervous system disorders, and diseases significantly (*p* < 0.05) regulated by these molecules and their associated molecular networks.

#### Ingenuity Pathway Analysis

Ingenuity Pathway Analysis (IPA) software (Qiagen Inc., MD, United States) has a cutting edge, up to date next generation knowledge base that consists of clarified scientific information from publications, databases, and other relevant resources (Jafri et al., 2019; Bahlas et al., 2020). Here, we applied the IPA to functionally annotate the protein clusters and identified biologically significant disease-specific pathways regulated by cathine and cathinone molecular targets. The putative molecular targets of cathine and cathinone was subjected to Core Analysis in the IPA to delineate biologically relevant canonical pathways, diseases, biological and pathological functions, upstream regulators, causal networks, and nondirectional unique networks, using the right-tailed Fisher Exact Test and Benjamini Hochberg Correction (BHC) for multiple testing (*p* < 0.05).

## Results

### Khat Extract and SKOV3 Cells Morphology

**FIGURE 1:**
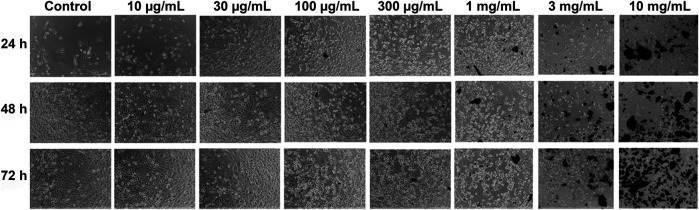
Representative phase-contrast images of the ovarian cancer cell line (SKOV3) following treatment with khat extracts at different concentrations (10, 30, 100, 300 μg/ml, 1, 3, 10 mg/ml) and different time points (24, 48, and 72 h). Compared to control, the SKOV3 cells exposed to khat extracts demonstrated cell growth inhibition and death with increasing concentration of the extract. Magnification 10x.

The untreated control of SKOV3 cells demonstrated normal attachment, morphology, growth, and proliferation. Treatment with different concentrations (10, 30, 100, and 300 μg/ml; 1, 3, and 10 mg/ml) of khat extracts for 24–72 h showed variable inhibition of SKOV3 cell growth and proliferation compared to the control (Figure 1). There were no changes in cell morphology or proliferation at lower concentrations compared to the control. However, higher concentrations of khat extracts, especially 1 mg/ml, 3 mg/ml, and 10 mg/ml and extended culture period (48 and 72 h), showed different morphological changes such as shrinkage in cell size, damage to cell membranes, and loss of cell adherence, culminating in cell death compared to control (Figure 1).

### Khat Extract and SKOV3 Cells Metabolic Activity

**FIGURE 2:**
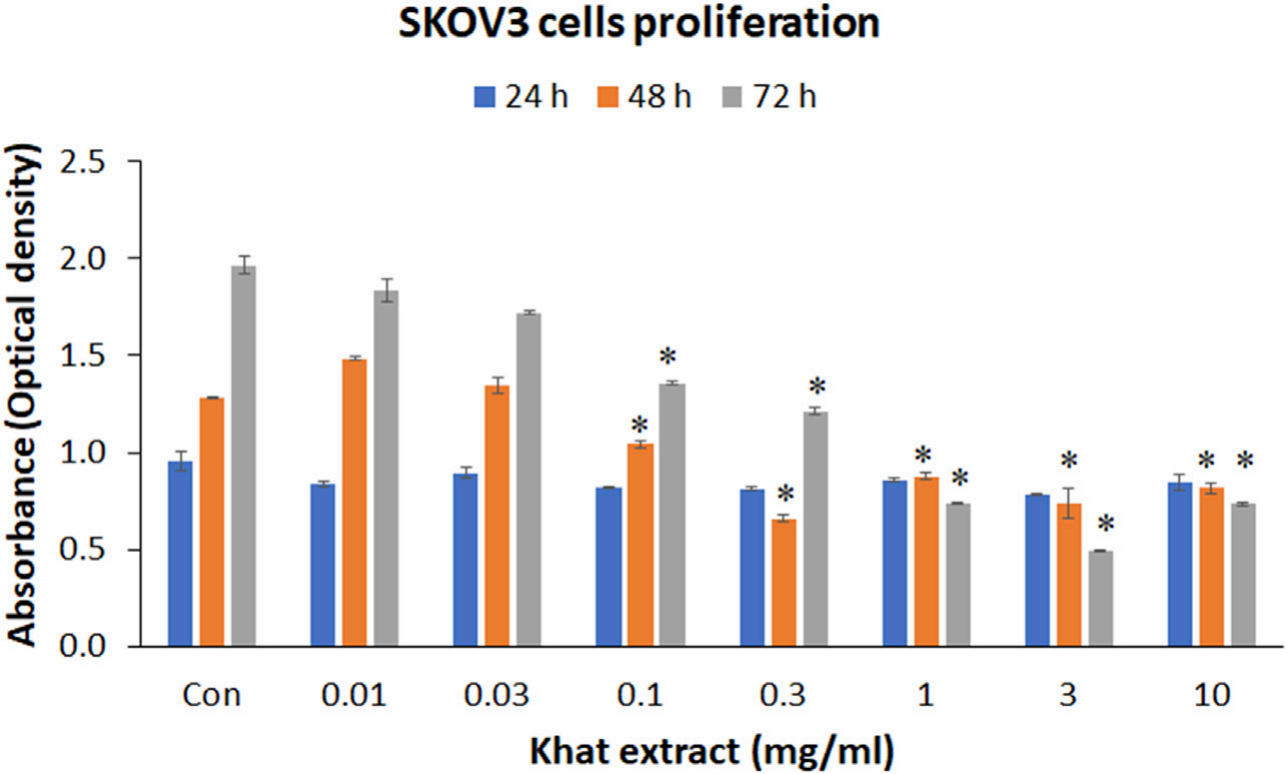
Cell metabolic activity (MTT assay) of SKOV3 following treatment with khat extracts at different concentrations (10, 30, 100, 300 µg/ml; 1, 3, 10 mg/ml) and different time points (24, 48, and 72 h). There was a decrease in cell metabolic activity reflecting cell proliferation with increasing concentration of the extract especially at 48 and 72 h compared to the control. Values are expressed as mean ± SEM of three different experiments.*Statistical significance (*p* < 0.05).

MTT assay demonstrated an indirect increase, reflecting the increase in cell numbers with extended duration of culture. However, following treatment with different concentrations (10, 30, 100, and 300 μg/ml; 1, 3, and 10 mg/ml) of khat extracts for 24–72 h, a mild to moderate decline in the metabolic activity of the SKOV3 cells was observed with most concentrations compared to the control (Figure 2). The observed reduction in metabolic activity after treatment for 24 h at various concentrations of khat extracts as stated above was 12.32, 6.34, 14.22, 14.92 10.13, 18.21, and 11.36%. These mean decreases compared to the control were statistically not significant (Figure 2).

At 48 h, the 10 and 30 μg/ml concentrations of khat extracts demonstrated an increase by 15.84 and 5.28%, respectively, compared to the control, these increases were not statistically significant. The rest of the concentrations (100 and 300 μg/ml; 1, 3, and 10 mg/ml) of khat extracts demonstrated a decrease by 18.68, 48.65, 31.55, 42.30, and 36.19% compared to the control. All these mean decreases in values were statistically significant (Figure 2).

Treatment of SKOV3 cells with khat extracts for 72 h demonstrated a decrease in the metabolic activity by 6.57, 12.52, 31.05, 38.40, 62.39, 74.85, and 62.72% compared to the control. All these mean decreases in values except for the 10 and 30 μg/ml concentrations were statistically significant (Figure 2).

### Khat Extract and Cell Cycle Assay

**FIGURE 3:**
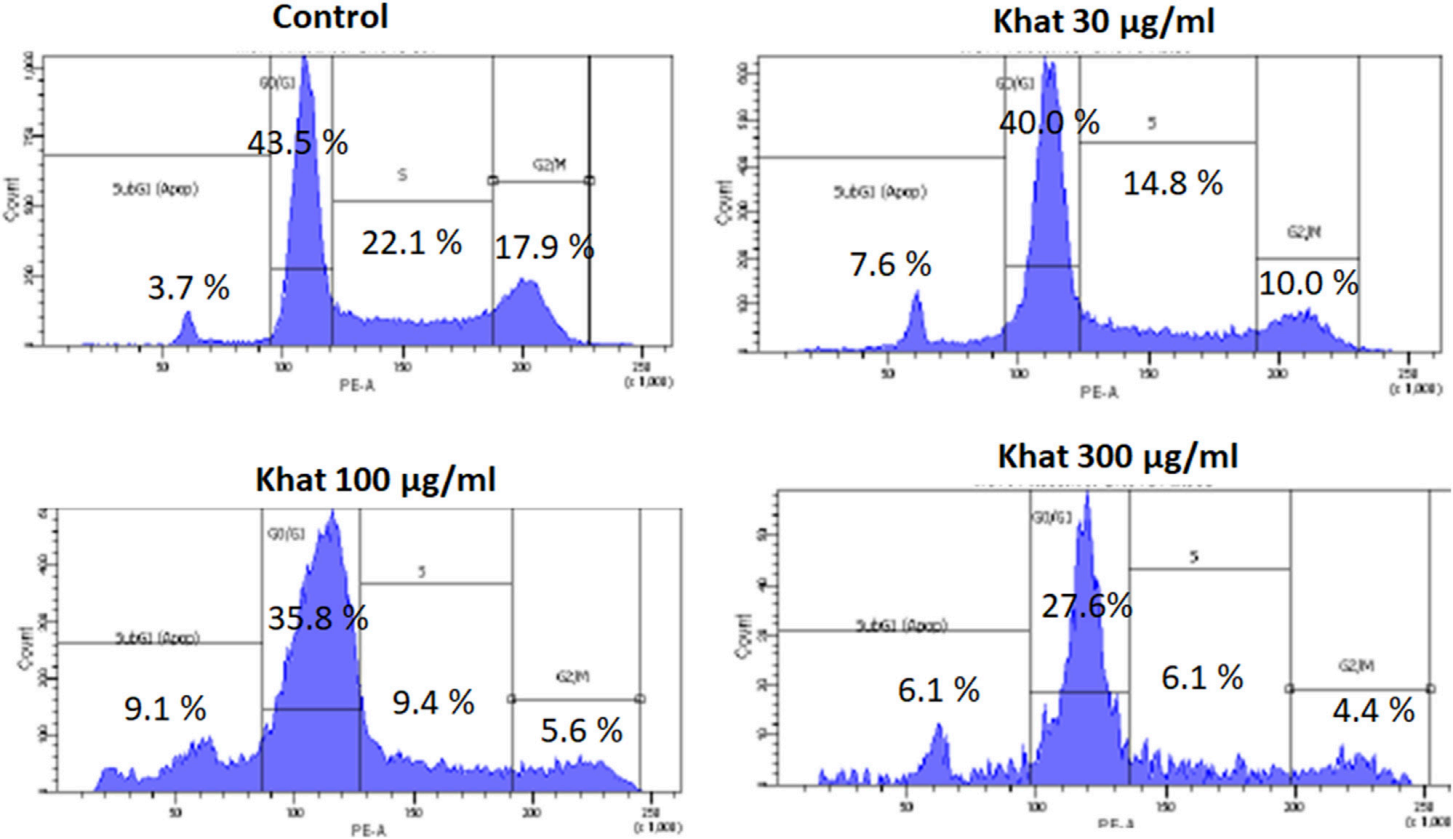
Cell cycle (Propidium iodide assay) of SKOV3 following treatment with khat extracts at different concentrations (30 μg/ml, 100 μg/ml, and 300 μg/ml) for 48 h. There was a mild increase in the sub-G1 phase of the cell cycle indicative of apoptosis.

The cell cycle (propidium iodide) assay evaluated after treatment of SKOV3 cells with 30, 100, and 300 μg/ml of khat extracts for 48 h demonstrated an increase in the sub-G1 SKOV3 cells’ population by 3.9, 5.4, and 2.4%, respectively, compared to the control in the representative histogram (Figure 3). The "S" phase of the cell cycle demonstrated a decrease by 7.8, 12.7, and 16.0%, respectively, compared to the control (Figure 3). The "G2M" phase of the cell cycle demonstrated a decrease by 7.9, 12.3, and 13.3%, respectively, compared to the control (Figure 3).

### Khat Extract and Cell Apoptosis Assay

**FIGURE 4:**
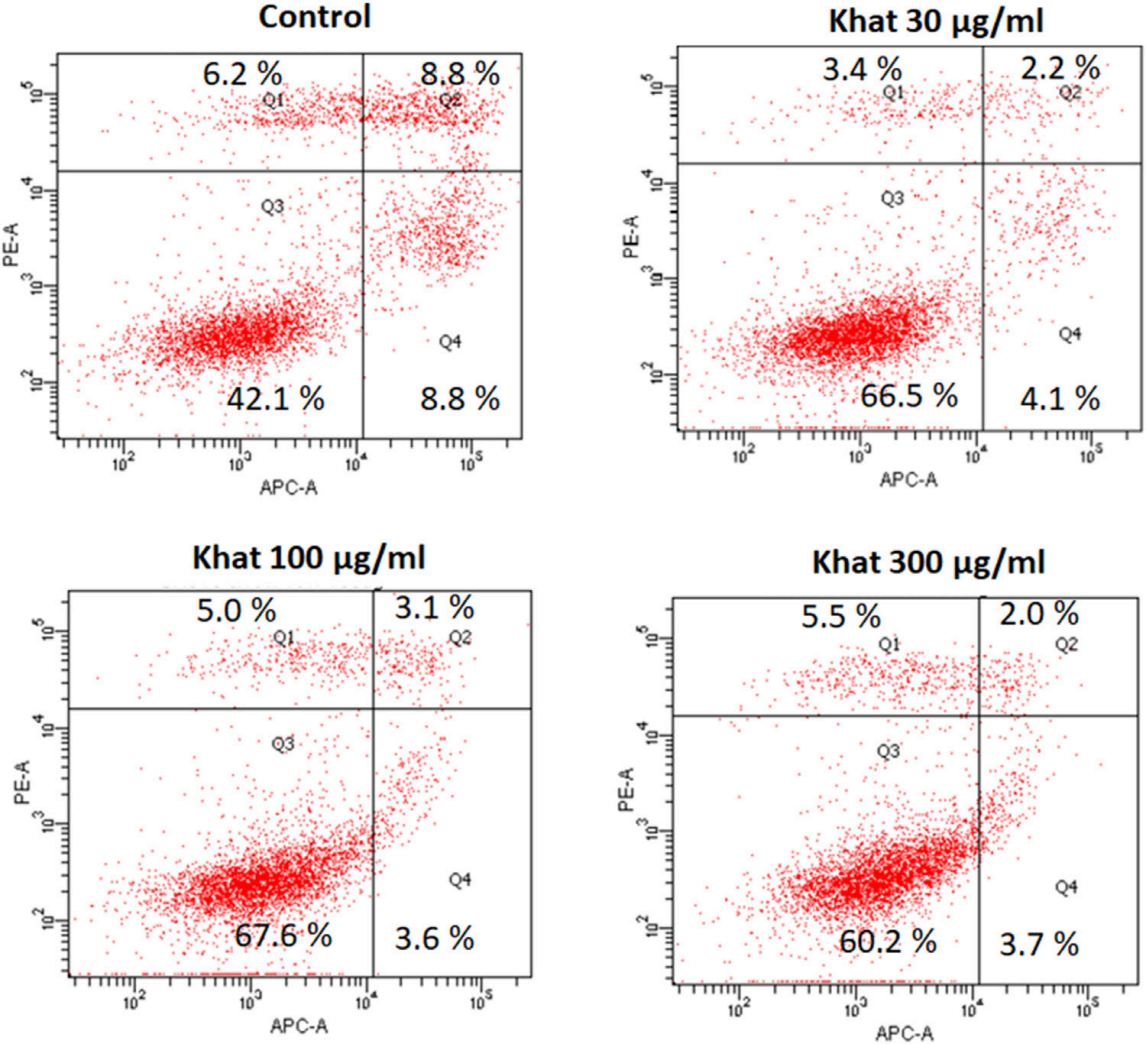
Apoptosis (annexin V-APC assay) of SKOV3 following treatment with khat extracts at different concentrations (30 μg/ml, 100 μg/ml, and 300 μg/ml) for 48 h. The control cells demonstrated more positive staining with annexin V-APC which showed a decreasing trend with increasing concentration of the khat extract.

The apoptosis (Annexin V-APC) assay evaluated with 30, 100, and 300 μg/ml of khat extracts for 48 h demonstrated a decrease in the apoptotic cells’ population compared to the control (Figure 4). The mean percentage values of the apoptotic cells were 2.2, 2.0, and 3.1% for the concentrations of 30, 100, and 300 μg/ml, respectively, compared to the control (Figure 4). The percentage of cells representing the cell debris population was increased by 24.4, 25.5, and 18.1% compared to the control (Figure 4).

### Khat Extract and Gene Expression Assay

**FIGURE 5:**
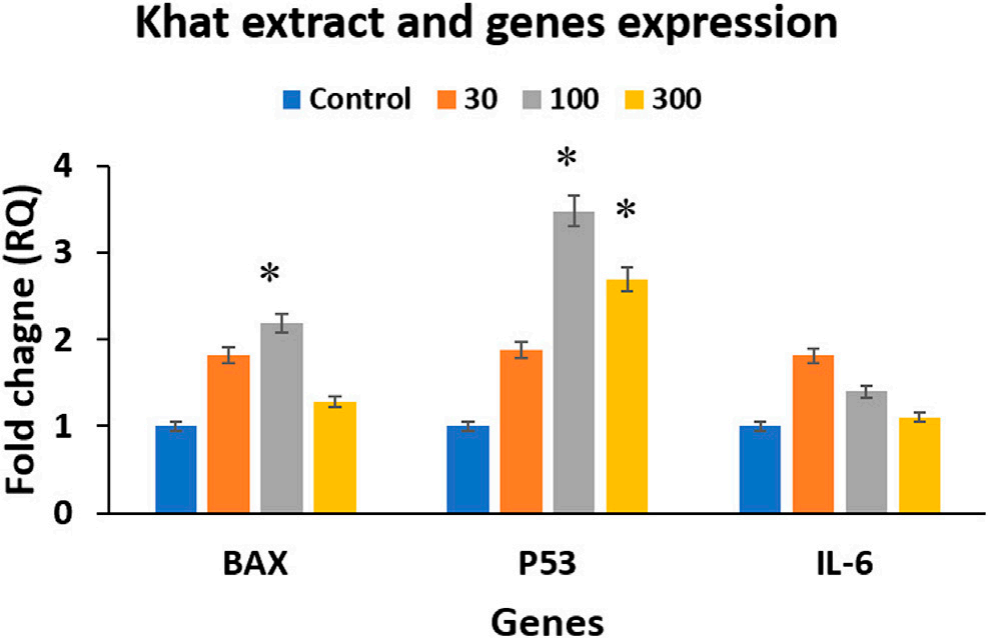
Gene expression analysis using RT-qPCR analysis of the treated and untreated SKOV3 cells showing BAX, p53, and IL-6 expression following treatment with 30, 100, and 300 μg/ml of khat extracts for 48 h. GAPDH was used as the internal control, and the data were quantified using the comparative 2^−ΔΔCt^ method. The values are expressed as mean ± SEM from triplicate samples of two independent experiments.

The quantitative RT-PCR analysis was carried out following treatment of SKOV3 cells with 30, 100 and 300 μg/ml of khat extracts for 48 h demonstrated a mild increase in the expression of both apoptosis-related BAX and p53 genes and inflammation-related IL-6 gene (Figure 5). The fold increases were as follows: BAX (0.81, 1.18, and 0.28); p53 (0.88, 2.48, and 1.69); and IL-6 (0.81, 0.40, and 0.10) with 30 μg/ml, 100 μg/ml, and 300 μg/ml of khat extracts, respectively, compared to the control (Figure 5). The proapoptotic BAX gene and the tumor suppressor p53 gene showed an overall increase compared to the control, although the higher concentration demonstrated a relative decrease. However, the inflammatory gene IL-6 was increased compared to the control; it demonstrated a decline with an increase in concentrations of khat extracts.

### Analysis of the Effects of Khat Extract by Immunostaining

**FIGURE 6:**
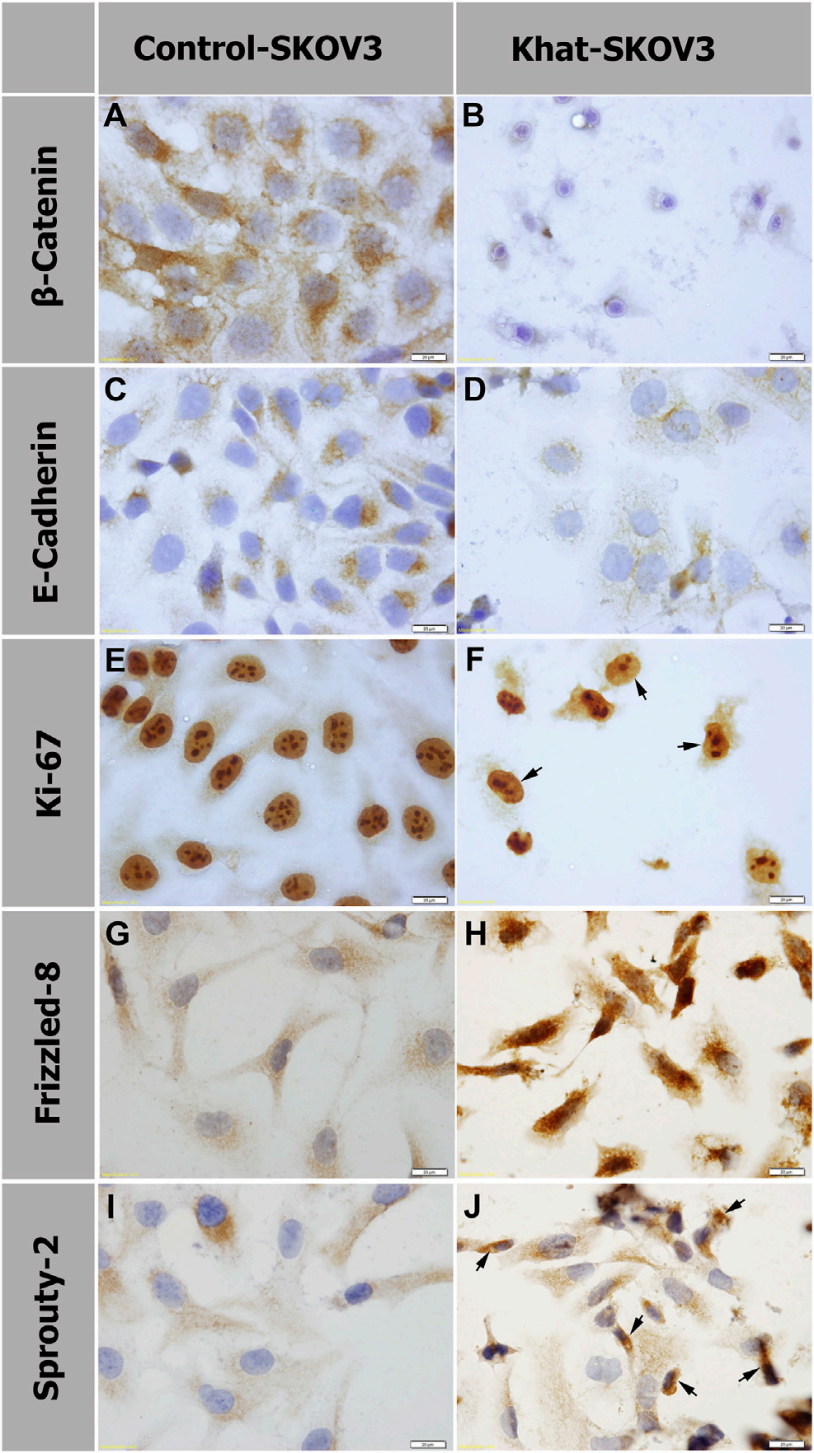
Protein expression analysis by immunostaining of β-catenin **(A, B)**, E-cadherin **(C , D)**, Ki-67 **(E, F)**, Frizzled-8 **(G, H)**, and Sprouty 2 **(I, J)** of SKOV3 cells treated with 700 μg/ml of khat extract alongside the untreated SKOV3 control cells. Panels **(B, D, F)** show downregulation of β-catenin, E-cadherin, and Ki-67, respectively, in comparison with the corresponding untreated control **(A, C, E)**. Panels **(H, J)** show increased expression of FZD8 and SPRY2, respectively, in comparison with the untreated control **(G, I)**. Arrows in **(F)** indicate SKOV3 khat-treated cells with a reduction in the nucleoli number. Arrows in **(J)** indicate SKOV3 khat-treated cells with an elevated SPRY2 expression.

The endogenous protein expression of several molecular markers was tested on khat SKOV3–treated cells at 700 μg/ml for 48 h alongside the untreated control cells by immunostaining. These markers were applied to specifically test the khat extract effects on important molecular and cellular signaling such as Wnt and FGF signaling, cellular adhesion, and cellular proliferation. The markers used were β-catenin and Frizzled-8 (Wnt signaling pathway molecules), E-cadherin (CAM or cell adhesion molecule), Sprouty-2 (FGF/MAP kinase signaling inhibitor), and Ki-67 (nonhistone nuclear protein marker for cell proliferation) (Figure 6 **)**. In SKOV3 khat extract treated cells, we observed downregulation of the protein expression of β-catenin, E-cadherin, and Ki-67 (Figures 6B,D,F) in comparison with their corresponding untreated controls (Figures 6A,C,E). A reduction in the nucleoli number was also observed which was demarcated by the Ki-67 expression (Figure 6F) in comparison with the untreated control cells (Figure 6E). A considerable upregulation of Frizzled-8 and Sprouty-2 expression (Figures 6H,J) in comparison with the untreated SKOV3 control (Figures 6G,I) was also observed.

### *In Silico* Analysis

#### Prediction of the Molecular Targets of Cathine, Cathinone, Catheduline K2, and Catheduline E5 Using SwissTargetPrediction

**FIGURE 7:**
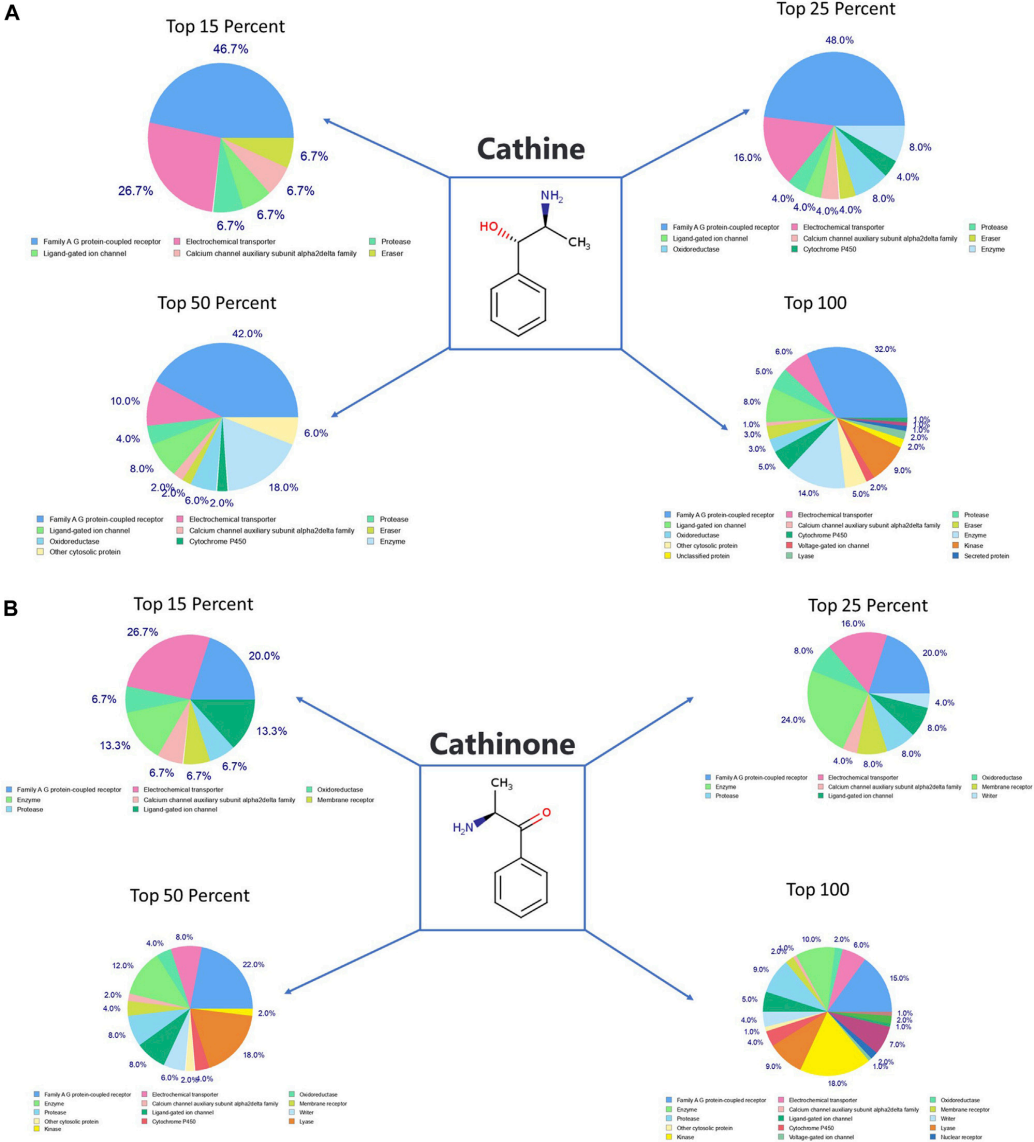
SwissTargetPrediction analysis for cathine **(A)** (PubChem CID 441457) and cathinone **(B)** (PubChem CID 62258) showing top targets for both components. Cathine and cathinone have the highest percentage of binding (32 and 15%, respectively) with family A G-protein-coupled receptors.

**FIGURE 8:**
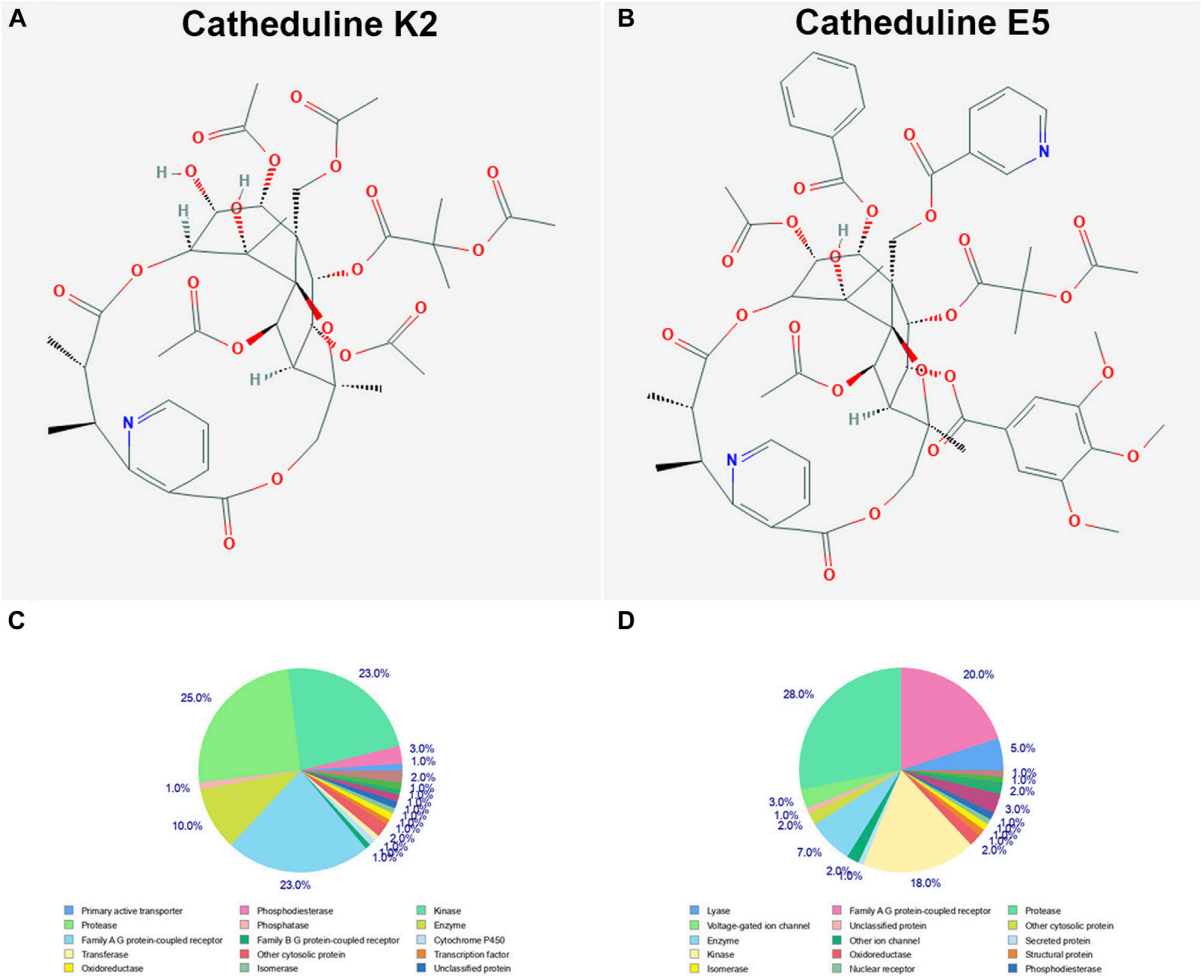
SwissTargetPrediction analysis of two members of the cathedulins khat constituents, catheduline K2 **(A)** (PubChem CID 101290208), and catheduline E5 **(B)** (PubChem CID 124201484) showing top 100 targets for both components **(C, D),** respectively. Both cathedulins have the potential to highly bind with the protease, kinase, and Family A G protein-coupled receptor in addition to other potential targets.

SwissTargetPrediction was performed for cathine (Figure 7A **)**, cathinone (Figure 7B), catheduline K2 (Figures 8A,C), and catheduline E5 (Figures 8B,D) using both canonical and isomeric Simplified Molecular Input Line Entry System (SMILES) codes (Supplementary Table S1) computed by OEChem (Version 2.1.5). The cathine and cathinone have the highest percentage of binding (32 and 15%, respectively) with family A G-protein coupled receptors (Figures 7A,B). For the cathedulins K2 and E5, one hundred top targets analysis showed that these compounds show high affinity to bind to the proteases, kinases, and Family A G protein-coupled receptor **(** Figures 8C,D). This is in addition to other targets such as cytochrome p450, nuclear receptor, and voltage-gated ion channel.

#### Over Representation Analysis (ORA) of the Molecular Targets of Cathine and Cathinone Using WebGestalt

**FIGURE 9:**
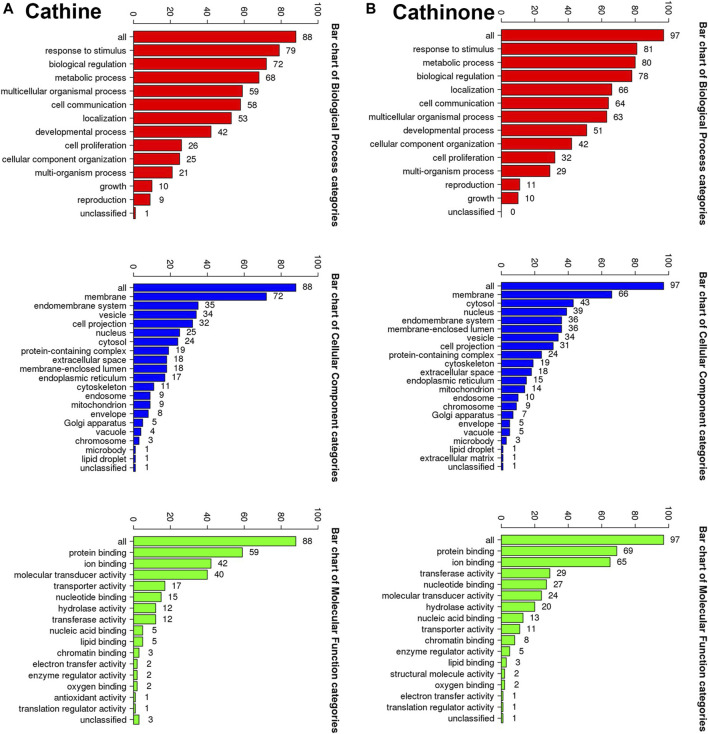
The GO Slim analysis. The summaries are based upon the 88 and 97 unique Entrez gene IDs for the putative protein targets of cathine **(A)** and cathinone **(B)**, respectively. Potential effects on the biological process (bars in red), cellular component (bars in blue) and molecular function (bars in green). The height of each bar characterizes the number of IDs in the user list and in the category.

All the putative molecular targets of cathine and cathinone were obtained using isomeric SMILES (Supplementary Table S1) as input molecules in wGSEA to perform the ORA. GO Slim Summary for cathine and cathinone molecular targets in humans displaying biological process, cellular component, and molecular function are shown in (Figure 9).

The putative target list contains 100 user IDs for either cathine or cathinone. 88 user IDs for cathine were unambiguously mapped to 88 unique Entrez gene IDs and 13 user IDs, while 97 user IDs for cathinone were unambiguously mapped to 97 unique Entrez gene IDs and three user IDs cannot be mapped to any Entrez gene ID. The GO Slim summary for cathine was based upon the 88 unique Entrez gene IDs. Among 88 unique Entrez gene IDs, 6 IDs were annotated to the selected functional categories as well as the reference list, which was used for the enrichment analysis (Figure 9A). Similarly, the GO Slim summary for cathinone was based upon the 97 unique Entrez gene IDs. Among 97 unique Entrez gene IDs, 9 IDs were also annotated in the same way (Figure 9B).

The reference lists consist of all mapped Entrez gene IDs from the selected platform genome. The ORA using OMIM, GLAD4U, and DisGeNET disease databases showed that the putative protein targets of both cathine and cathinone significantly (Supplementary Figure S2, indicated by the false discovery rate (FDR ≤ 0.05) in dark blue) regulate many diseases and disorders such as schizophrenia, Alzheimer’s disease, mental depression, and other major depressive disorders, anxiety disorders, and migraine disorders.

#### Identification of Cathine and Cathinone-Induced Molecular Targets in Cell Proliferation Disorders, Ovarian Diseases, and Psychiatric Disorders

The Open Targets Platform was used to determine the association between diseases with the putative molecular targets of both cathine and cathinone (Supplementary Figure S1). Our findings showed that 879 types of cell proliferation disorders (Supplementary Table S2), 13 different types of ovarian diseases (Supplementary Table S3), and 322 types of psychiatric disorders (Supplementary Table S4) were significantly (*p* < 0.05) affected by the molecular targets of cathine. The 13 types of ovarian diseases significantly (*p* < 0.05) regulated by cathine were noticeably cancer-related diseases.

Additionally, our findings showed that 1,156 types of cell proliferation disorders (Supplementary Table S5), 43 different types of ovarian diseases (Supplementary Table S6), and 408 types of psychiatric diseases (Supplementary Table S7) were significantly (*p* < 0.05) affected by the molecular targets of cathinone. The 43 types of ovarian diseases significantly (*p* < 0.05) regulated by cathinone were predominantly related to ovarian cancer disease, ovarian insufficiency, ovarian dysfunction leading to infertility, rare female infertility due to an anomaly of the ovarian function of genetic origin, osteosclerosis-ichthyosis-premature ovarian failure, and ovarian endometriosis. (Supplementary Table S6).

#### Ingenuity Pathway Analysis of the Putative Protein Targets of Cathine and Cathinone

We used the IPA to decode the canonical pathways, upstream regulators, causal functions, diseases and biofunctions, pathological functions, and nondirectional unique networks that are significantly impacted by both cathine and cathinone. The IPA core analysis of the putative molecular targets of cathine (Supplementary Table S8) and cathinone (Supplementary Table S9) revealed that canonical pathways such as G-protein coupled receptor signaling, dopamine receptor signaling, serotonin receptor signaling, CREB-signaling in neurons, Wnt signaling, FGF signaling, IL-6 signaling, ERK/MAPK signaling, endometrial cancer signaling, and cell cycle were significantly affected (*p* < 0.05). Furthermore, the diseases and biofunctions such as psychological disorders and many neurological diseases were potentially regulated (*p* < 0.05) by cathine (Supplementary Table S10) and cathinone (Supplementary Table S11).

Interestingly, the IPA core analysis identified 250 upstream regulators of cathine to target CXCL8 (Interleukin-8), and 43 regulators to target PLAU and its receptor PLAUR (Supplementary Table S12). On the other hand, the analysis identified 33 upstream regulators of cathinone (not the cathine) to target MMP2 and 62 regulators to target PLAU and its receptor PLAUR (Supplementary Table S13).

The IPA analysis identified several upstream micro-RNA regulators. For the cathine, these were miR-16-1-3p, miR-30, miR-30c-5p, miR-31-5p, miR-146a-5p, mir-204, miR-373, miR-424-3p, miR-511-5p, and miR-542-3p (Supplementary Table S12). For the cathinone, these were: miR-9, miR-9-5p, miR-30c-5p, miR-31, miR-34a-5p, miR-103, miR-222-5p, miR-296, miR-451a, miR-491-5p, miR-1180, miR-1275, and miR-1285-3p (Supplementary Table S13).

## Discussion

Khat plant is a widespread stimulant which is recreationally munched by many people in Africa, Asia, Europe, and the United States with an estimate of more than 20 million users (El-Menyar et al., 2015). Many studies have shown that khat induces a series of adverse effects during embryonic development and illnesses in adulthood. These include teratogenicity, cancer, and adverse effects on the nervous, cardiovascular, digestive, genitourinary, reproductive, metabolic, and endocrine systems (reviewed by Wabe, 2011). In this study, we evaluated the effects of khat on the human ovarian adenocarcinoma SKOV3 cell line. We detected several cellular and molecular adverse effects, including shrinkage in the cell size, damage to the cell membrane, loss of cell adherence, cell death, metabolic decline, decrease in the "S" and "G2" cell cycle phases, and decrease in the apoptotic cells’ population. Similar effects of inhibition of cell proliferation and cell growth, shrinkage of cells and increased apoptosis were shown after khat extracts treatment in human hepatocytes HepG2 (Taha et al., 2014), rat cardiomyoblasts H9c2 (Mohan et al., 2016), and Madin–Darby bovine kidney cell line (Ageely et al., 2016). It was also previously shown that khat induced a reduction in cell viability and apoptosis in other different cell lines such as L02 human hepatic cell line (Abid et al., 2013) and human breast cancer cell line MDA-MB-231 (Lu et al., 2017). It has been shown that khat induced apoptosis through a mechanism involving activation of caspase-1, -3, and -8 (Dimba et al., 2004).

We tested the khat effects on apoptosis, tumorigenesis, and inflammation using BAX, p53, and IL-6, respectively, and showed an overall increase in the expression of these important genes. Our results agree with several studies that tested the effects of khat on the expression of these markers but using different cell lines. Abid et al. (2013) showed an increase in BAX expression after khat extracts treatment to the human liver cell line L02. An increase of p53 expression and a G1-phase arrest was previously reported after the khat extracts treatment of human oral keratinocytes and oral fibroblasts (Lukandu et al., 2008). An increase in the IL-6 expression in the brain tissue was previously reported after khat extracts treatment *in vivo* in mice (Ali et al., 2015).

Khat extracts treatment in rats showed stress-related effects on the ovaries due to an imbalance between ROS and the production of antioxidants (Arafa et al., 2019). Similar ROS inhibition induced by khat extracts treatment was reported using murine monocytic macrophages RAW 264.7 cell line (Abdelwahab et al., 2018). It has also been reported that khat induces intracellular ROS in the human fetal hepatocyte L02 cell line resulting in consecutive activation of JNK and ERK signaling pathways (Abid et al., 2013). This, in turn, decreased cell viability and increased apoptosis. In the current study, the effects of khat extracts treatment on ROS were not tested; however, ROS may have been similarly affected in SKOV3 cells.

We aimed to analyze possible molecular signaling pathways after khat extracts treatment to decipher the involved mechanisms of action. Among several signaling pathways, Wnt signaling (represented by β-catenin and FZD8 expression), FGF signaling (represented by SPRY2 expression, FGF negative regulator), and cellular adhesion (represented by E-Cadherin) were tested using respective antibodies against these markers. Despite their crucial roles during many cellular events, these signaling pathways have not been previously analyzed following khat extracts treatment. We showed in the current study that β-catenin expression was severely reduced after khat extracts treatment. This suggests that canonical Wnt signaling through β-catenin was modulated. Interestingly, we also showed that FZD8 (Wnt receptor) expression was strongly elevated in comparison with the untreated SKOV3 control cells. It was previously shown that elevated FZD8 expression was linked with the airway proinflammation induction and associated with chronic bronchitis (Spanjer et al., 2016). As mentioned above, we showed by RT-qPCR an increase in the proinflammation IL-6 expression which could be the trigger for FZD8 expression upregulation. This explanation is supported by some reported evidence linking Wnt/β-catenin signaling with both anti-inflammation and proinflammation functions (Ma and Hottiger, 2016). We also showed that SPRY2 expression was upregulated following treatment with khat extracts. SPRY2 is a negative regulator of FGF signaling, so upregulation of its expression would lead to blocking of the FGF signaling through a negative feedback loop. Compromising FGF signaling might explain some of the cellular damage obtained in our results. We also observed a reduction in E-cadherin expression. It has been previously reported that SPRY2 overexpression inhibited the induction of the transcriptional repressor E-cadherin in the SKOV3 ovarian adenocarcinoma cell line (Cheng et al., 2016).

Our *in silico* results from various analyses showed that cathine, cathinone, catheduline K2, and catheduline E5 potentially induce several neurological and psychological diseases and symptoms. Our findings agree with earlier results reported by other studies (Odenwald et al., 2005; Hoffman and Al’absi, 2010; El-Setouhy et al., 2016). However, potential induction of several neural disorders we report here by the khat constituents such as developmental disorder of mental health, neurodevelopmental disorder with epilepsy, motor developmental delay, macrocephaly, developmental delay with seizures, and developmental delay associated with premature aging appearance (*p* < 0.05) (Supplementary Tables S4, S7) have not been previously reported. Further experimental validation of these results would complement the *in silico* analysis and is highly recommended.

Additionally, the IPA analysis results (Supplementary Tables S2, S5, S8, S9) supported our immunostaining findings which showed that the khat extract affected cell proliferation, Wnt signaling, FGF signaling, and cell adhesion in SKOV3 cells. Besides, the IPA analysis showed that upstream targets of the cathine and cathinone abundantly target MMP2, PLAU, and its receptor PLAUR, and IL-8. It has been previously shown that MMP2 functions as an early marker for ovarian cancer metastasis (Kenny and Lengyel, 2009). The urokinase plasminogen activator (PALU) was shown to play an important role during ovulation in animal models (Ogiwara et al., 2015). Interestingly, IL-8 was shown to increase cell proliferation and correlate with increased MMP2 expression in ovarian cancer (Wang et al., 2012).

The IPA analysis identified several members of the micro-RNAs as potential upstream regulators of the cathine and cathinone. These micro-RNAs have previously been reported to have different important roles in ovarian cancer regulation, prognosis, and/or diagnosis (Alshamrani, 2020; Aziz et al., 2020; Ferreira et al., 2020). Hence, our present analysis could provide potential directions for future studies on the further elucidation of these micro-RNAs’ regulations of the cellular and molecular events induced by khat. In summary, our study identifies several crucial molecular signaling pathways mediated by khat extracts treatment and not been previously identified.

## Conclusions

We examined the cellular and molecular side effects of khat extracts on the human ovarian adenocarcinoma SKOV3 cell line, aiming mainly at deciphering the implicated signaling pathways. We showed by several *in vitro* assays that khat extracts affects the cellular integrity of SKOV3, including size, membrane, metabolic activity, proliferation, and survival. At the gene and protein levels, expression of BAX, p53, IL-6, FZD8, and SPRY2 was increased while β-catenin, E-cadherin, and Ki-67 was decreased. Our *in silico* analysis revealed that khat extracts’ major constituents namely cathine, cathinone, and cathedulins are potentially associated with Alzheimer's, schizophrenia, depression, anxiety, and ovarian cancer. Signaling pathways of CREB, Wnt, FGF, IL-6, and ERK/MAPK were among other pathways significantly affected. Besides, the upstream regulators including IL-8, MMP2, PLAU, and an array of micro-RNAs were potentially involved in the khat signaling.

## Data Availability

The original contributions presented in the study are included in the article/Supplementary Material, and further inquiries can be directed to the corresponding author.
