# Supplementary material for: Unraveling the Catha edulis Extract Effects on the Cellular and Molecular Signaling in SKOV3 Cells
Source: Front Pharmacol. 2021 May 10;12:666885. doi: 10.3389/fphar.2021.666885 (PMC8141790; doi:10.3389/fphar.2021.666885)
Supplement: Supplementary file 3 [file Table1.pdf]

Table S1: The putative molecular targets of the khat constituents cathine and cathinone, obtained based on both canonical and isomeric SMILES from the PubChem Database. The target list also shows the associated target classes identified by Swiss Target Prediction.

SwissTargetPrediction				
Cathine			Cathinone	
**No.**	**Uniprot ID**	**Target Class**	**Uniprot ID**	**Target Class**
1	P08913	Family A G protein-coupled receptor	P25103	Family A G protein-coupled receptor
2	P18089	Family A G protein-coupled receptor	P31645	Electrochemical transporter
3	P35348	Family A G protein-coupled receptor	Q01959	Electrochemical transporter
4	P31645	Electrochemical transporter	P23975	Electrochemical transporter
5	Q96RJ0	Family A G protein-coupled receptor	Q96RJ0	Family A G protein-coupled receptor
6	Q01959	Electrochemical transporter	P27338	Oxidoreductase
7	P23975	Electrochemical transporter	P23141	Enzyme
8	P28223	Family A G protein-coupled receptor	O00748	Enzyme
9	P15144	Protease	P28223	Family A G protein-coupled receptor
10	P46098	Ligand-gated ion channel	Q05940	Electrochemical transporter
11	Q05940	Electrochemical transporter	P54289	Calcium channel auxiliary subunit alpha2delta family
12	P54289	Calcium channel auxiliary subunit alpha2delta family	Q03405	Membrane receptor
13	O60341	Eraser	Q6V1X1	Protease
14	P28221	Family A G protein-coupled receptor	P32297 P30926	Ligand-gated ion channel
15	P14416	Family A G protein-coupled receptor	P43681	Ligand-gated ion channel
16	P21397	Oxidoreductase	P29475	Enzyme
17	P27338	Oxidoreductase	P35228	Enzyme
18	P07550	Family A G protein-coupled receptor	P29474	Enzyme
19	Q9H3N8	Family A G protein-coupled receptor	Q99720	Membrane receptor
20	P25103	Family A G protein-coupled receptor	P11086	Enzyme
21	Q969F8	Family A G protein-coupled receptor	Q92831	Writer
22	P11509	Cytochrome P450	P08253	Protease
23	P11086	Enzyme	Q969F8	Family A G protein-coupled receptor
24	Q16853	Enzyme	P21397	Oxidoreductase
25	P08588	Family A G protein-coupled receptor	P25021	Family A G protein-coupled receptor
26	P35228	Enzyme	Q9H3N8	Family A G protein-coupled receptor
27	P30926 P43681	Ligand-gated ion channel	Q9Y2I1	Other cytosolic protein
28	P30926 Q15822	Ligand-gated ion channel	P32297 P17787	Ligand-gated ion channel
29	P00390	Oxidoreductase	P33261	Cytochrome P450
30	P18825	Family A G protein-coupled receptor	P11509	Cytochrome P450
31	P28335	Family A G protein-coupled receptor	P30542	Family A G protein-coupled receptor
32	Q9H228	Family A G protein-coupled receptor	P36544	Ligand-gated ion channel
33	O95977	Family A G protein-coupled receptor	P43166	Lyase
34	Q99500	Family A G protein-coupled receptor	P00915	Lyase
35	P21453	Family A G protein-coupled receptor	P23280	Lyase
36	P16662	Enzyme	O43570	Lyase
37	P29475	Enzyme	Q9ULX7	Lyase
38	Q9GZQ4	Family A G protein-coupled receptor	Q16790	Lyase
39	Q9UHL4	Protease	Q9Y2D0	Lyase
40	Q13126	Enzyme	P35218	Lyase

41	Q01650	Electrochemical transporter	Q8N1Q1	Lyase
42	P08908	Family A G protein-coupled receptor	P15144	Protease
43	P23526	Enzyme	Q96LA8	Writer
44	P43681	Ligand-gated ion channel	Q13639	Family A G protein-coupled receptor
45	P00491	Enzyme	P18825	Family A G protein-coupled receptor
46	P35462	Family A G protein-coupled receptor	Q86X55	Writer
47	P52732	Other cytosolic protein	P08913	Family A G protein-coupled receptor
48	Q16831	Enzyme	Q9UHL4	Protease
49	O96020 P24941 P24864	Other cytosolic protein	P14416	Family A G protein-coupled receptor
50	P50750 O60563	Other cytosolic protein	Q13464	Kinase
51	P36639	Enzyme	Q01650	Electrochemical transporter
52	P00749	Protease	Q9Y5N1	Family A G protein-coupled receptor
53	P27487	Protease	O75116	Kinase
54	P35368	Family A G protein-coupled receptor	P00749	Protease
55	P22460	Voltage-gated ion channel	P28335	Family A G protein-coupled receptor
56	P51812	Kinase	P35462	Family A G protein-coupled receptor
57	P11940	Unclassified protein	P48048	Voltage-gated ion channel
58	P36544	Ligand-gated ion channel	P10827	Nuclear receptor
59	P10635	Cytochrome P450	P10828	Nuclear receptor
60	P11712	Cytochrome P450	P32297	Ligand-gated ion channel
61	P08684	Cytochrome P450	Q9UGN5	Enzyme
62	P33261	Cytochrome P450	Q9UBN7	Eraser
63	Q13224	Ligand-gated ion channel	P19801	Enzyme
64	Q05586 Q13224	Ligand-gated ion channel	Q86TI2	Protease
65	Q15125	Enzyme	Q9BY41	Eraser
66	Q9ULX7	Lyase	Q13547	Eraser
67	P23141	Enzyme	Q96KB5	Kinase
68	O00748	Enzyme	P11712	Cytochrome P450
69	P24385 P11802	Kinase	P08684	Cytochrome P450
70	P06493 P14635	Other cytosolic protein	Q9UIF9	Reader
71	P24864 P24941	Kinase	P21964	Transferase
72	O14757	Kinase	Q9P1W9	Kinase
73	P35372	Family A G protein-coupled receptor	P07384	Protease
74	P41143	Family A G protein-coupled receptor	Q6QHF9	Enzyme
75	Q9UKL0 O60341	Eraser	P23458	Kinase
76	P24941 P78396 P20248	Other cytosolic protein	O60674	Kinase
77	P10145	Secreted protein	Q05655	Kinase
78	Q4U2R8	Electrochemical transporter	Q02156	Kinase
79	P11926	Lyase	Q15139	Kinase
80	Q99720	Membrane receptor	P00491	Enzyme
81	Q13639	Family A G protein-coupled receptor	Q12884	Protease
82	P29274	Family A G protein-coupled receptor	P51817	Kinase
83	P29275	Family A G protein-coupled receptor	Q86V86	Kinase
84	P50579	Protease	Q92769	Eraser
85	Q8NET8	Voltage-gated ion channel	P04818	Transferase
86	P37058	Enzyme	O60341	Eraser
87	P50750	Kinase	Q9NR22	Writer
88	P41145	Family A G protein-coupled receptor	P41595	Family A G protein-coupled receptor
89	Q13418	Kinase	O00311	Kinase

90	O75899 Q9UBS5	Family C G protein-coupled receptor	P31751	Kinase
91	B2RXH2	Eraser	P28838	Protease
92	P06241	Kinase	Q8IXJ6	Eraser
93	P41146	Family A G protein-coupled receptor	Q96EB6	Eraser
94	P06239	Kinase	Q96PY6	Kinase
95	P14867 P47870 P18507	Ligand-gated ion channel	O75899 Q9UBS5	Family C G protein-coupled receptor
96	P21554	Family A G protein-coupled receptor	P49841	Kinase
97	P34972	Family A G protein-coupled receptor	P06241	Kinase
98	P63316 P45379 P19429	Unclassified protein	P06239	Kinase
99	O00311	Kinase	P46059	Electrochemical transporter
100	P41595	Family A G protein-coupled receptor	Q02750	Kinase
