# Supplementary material for: Unraveling the Catha edulis Extract Effects on the Cellular and Molecular Signaling in SKOV3 Cells
Source: Front Pharmacol. 2021 May 10;12:666885. doi: 10.3389/fphar.2021.666885 (PMC8141790; doi:10.3389/fphar.2021.666885)
Supplement: Supplementary file 5 [file Image2.pdf]

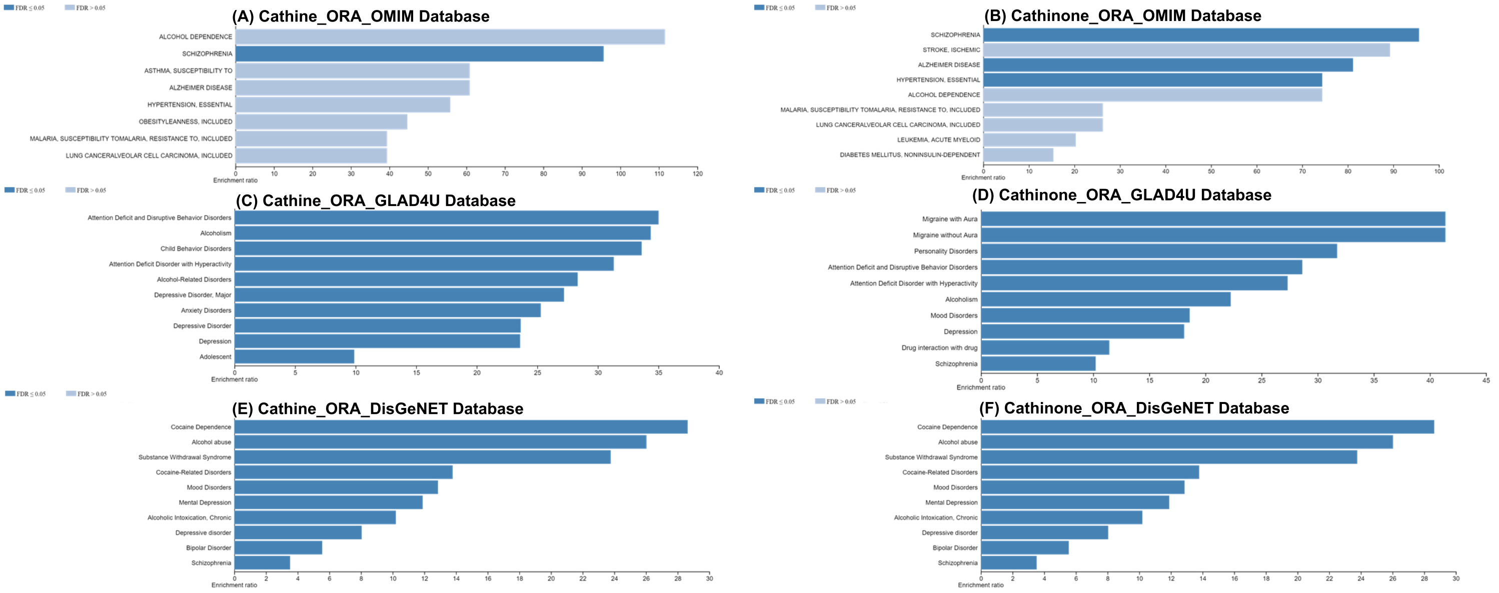

Figure S2: Over Representation Analysis (ORA) of the putative protein targets of cathine and cathinone using OMIM, GLAD4U, and DisGeNET databases. Many diseases induced or affected by the khat constituents are indicated by bars of the faults discovery rate (FDR) either in dark blue (p≤0.05) or light blue (p≥0.05).
