# Supplementary material for: Unraveling the Catha edulis Extract Effects on the Cellular and Molecular Signaling in SKOV3 Cells
Source: Front Pharmacol. 2021 May 10;12:666885. doi: 10.3389/fphar.2021.666885 (PMC8141790; doi:10.3389/fphar.2021.666885)
Supplement: Supplementary file 7 [file DataSheet1.pdf]

Captions for Supplementary Materials

Table S1: The putative molecular targets of the khat constituents cathine and cathinone, obtained based on both canonical and isomeric SMILES from the PubChem Database. The target list also shows the associated target classes identified by SwissTargetPrediction.

Table S2: Identification of cathine-induced molecular targets in cell proliferation showing 879 types of disorders ($p<0.05$).

Table S3: Identification of cathine-induced molecular targets in ovarian diseases showing 13 types of disorders ($p<0.05$).

Table S4: Identification of cathine-induced molecular targets in psychiatric disorders showing 322 types ($p<0.05$).

Table S5: Identification of cathinone-induced molecular targets in cell proliferation showing 1156 types of disorders ($p<0.05$).

Table S6: Identification of cathinone-induced molecular targets in ovarian diseases showing 43 types ($p<0.05$).

Table S7: Identification of cathinone-induced molecular targets in psychiatric disorders showing 408 types ($p<0.05$).

Table S8: Ingenuity Pathway Analysis of the putative protein targets of the cathine showing the affected canonical pathways.

Table S9: Ingenuity Pathway Analysis of the putative protein targets of the cathinone showing the affected canonical pathways.

Table S10: Ingenuity Pathway Analysis of the putative protein targets of the cathine showing the regulated diseases and bio functions.

Table S11: Ingenuity Pathway Analysis of the putative protein targets of the cathinone showing the regulated diseases and bio functions.

Table S12: Ingenuity Pathway Analysis of the putative protein targets of the cathine showing the upstream regulators. IL-8 (CXCL8), PLAU and PLAUR are the most targeted molecules by the upstream regulators.

Table S13: Ingenuity Pathway Analysis of the putative protein targets of the cathinone showing the upstream regulators. MMP2, PLAU and PLAUR are the most targeted molecules by the upstream regulators.

Figure S1: In silico analysis flowchart for cathine and cathinone.

Figure S2: Over Representation Analysis (ORA) of the putative protein targets of cathine and cathinone using OMIM, GLAD4U, and DisGeNET databases. Many diseases induced or affected by the khat constituents are indicated by bars of the faults discovery rate (FDR) either in dark blue ($p\leq0.05$) or light blue ($p\geq0.05$).
