# Supplementary figures and images for: Unraveling the Catha edulis Extract Effects on the Cellular and Molecular Signaling in SKOV3 Cells
Source: Front Pharmacol. 2021 May 10;12:666885. doi: 10.3389/fphar.2021.666885 (PMC8141790; doi:10.3389/fphar.2021.666885)

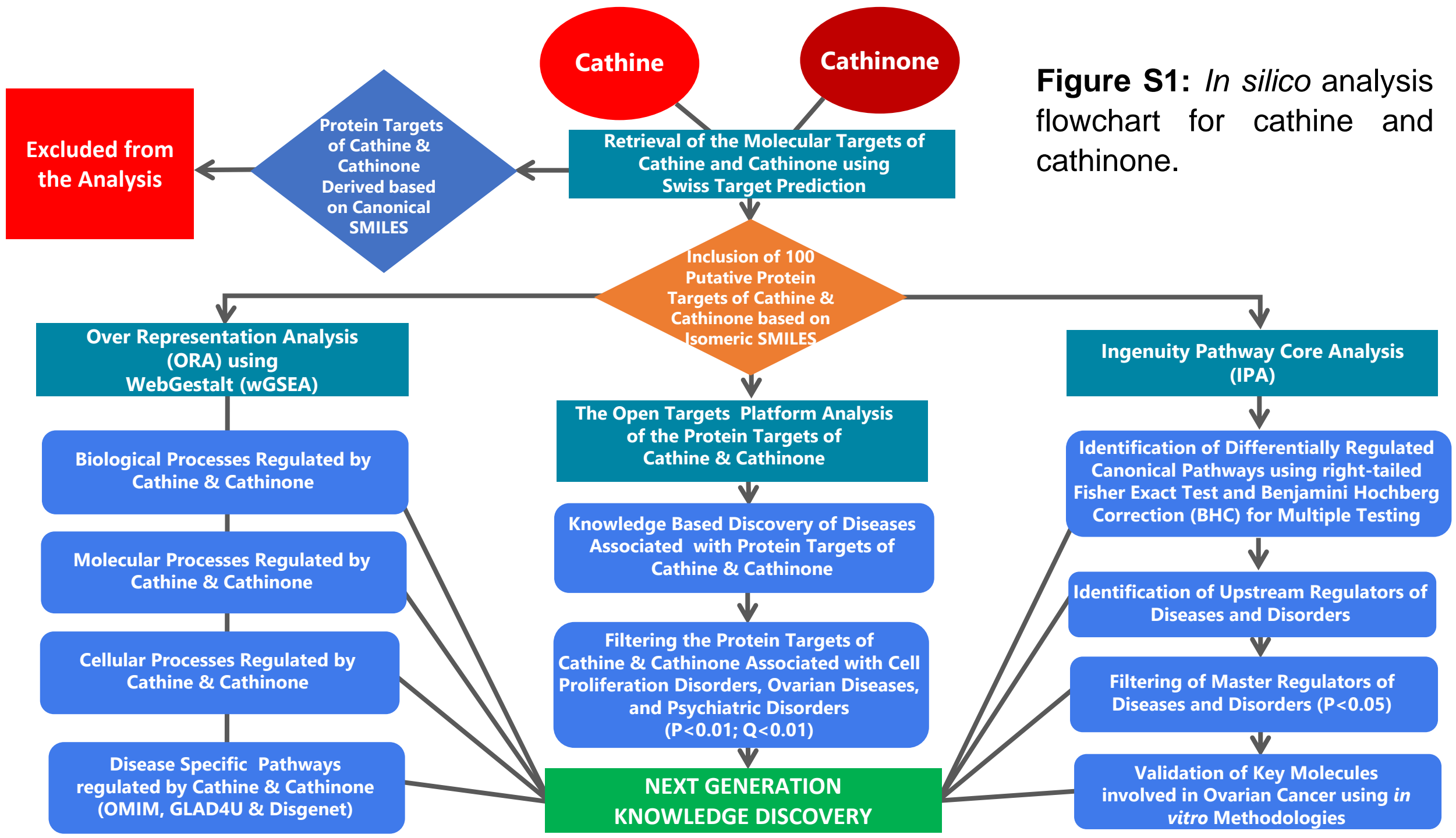

Supplement: Supplementary file 16 [file Image1.pdf]
